# Nanostructure and nanoindentation study of pulse electric-current sintered TiB_2_–SiC–C_f_ composite

**DOI:** 10.1038/s41598-022-27186-8

**Published:** 2023-01-07

**Authors:** Mohammadreza Shokouhimehr, Seyed Ali Delbari, Abbas Sabahi Namini, Ehsan Taghizadeh, Sunghoon Jung, Jin Hyuk Cho, Quyet Van Le, Joo Hwan Cha, Soo Young Kim, Ho Won Jang

**Affiliations:** 1 Department of Materials Science and Engineering, Research Institute of Advanced Materials, Seoul National University, Seoul, 08826 Republic of Korea; 2 Department of Engineering Sciences, Faculty of Advanced Technologies, University of Mohaghegh Ardabili, Ardabil, Iran; 3 Department of Mechanical and Aerospace Engineering, University of California, 420 Westwood Plaza, Los Angeles, CA 90095 USA; 4 Department of Nano-bio Convergence, Korea Institute of Materials Science, Changwon, 51508 Republic of Korea; 5 Department of Materials Science and Engineering, Institute of Green Manufacturing Technology, Korea University, 145, Anam-Ro Seongbuk-Gu, Seoul, 02841 Republic of Korea; 6 Innovative Enterprise Cooperation Center, Korea Institute of Science and Technology, Hwarangro 14-Gil, Seongbuk-Gu, Seoul, Republic of Korea

**Keywords:** Ceramics, Structural properties

## Abstract

A carbon-fiber (C_f_) doped TiB_2_–SiC composite was prepared and investigated to determine its densification behavior, micro/nanostructural properties, and mechanical characteristics. TiB_2_–25 vol% SiC–2 wt% C_f_ was prepared at 40 MPa and 1800 °C for 7 min using the pulsed electric-current sintering technique, and a relative density of 98.5% was realized. The as-sintered composite was characterized using advanced techniques, e.g*.*, X-ray diffractometry, energy-dispersive X-ray spectroscopy, X-ray photoelectron spectroscopy, field-emission scanning electron microscopy, high-resolution transmission electron microscopy, field-emission electron probe micro-analysis, and nanoindentation. The C_f_ additive could remove the surface oxide layers from the TiB_2_ and SiC domains, thus transforming them into TiB_2_ and SiC. According to micro/nanostructural studies, C_f_ could not retain its initial structure and was eventually converted into graphite nanosheets. In addition, the prepared composite was examined using the nanoindentation technique, and the following results were obtained for the calculated hardness, elastic modulus, and stiffness values: TiB_2_ > SiC > TiB_2_/SiC interface.

## Introduction

Titanium diboride (TiB_2_), a widely applicable ultra-high-temperature ceramic, possesses outstanding properties, including a superior melting point, high electrical conductivity, high thermal conductivity, and excellent hardness^1–5^. This ceramic's unique properties make it appropriate for a variety of advanced applications, including turbine blades, cutting tools, engine valves, electrodes for electro-discharge machining, and cathodes for aluminum electrolysis^6–9^. However, its strong covalent bonding and low self-diffusion coefficient hamper its sinterability, particularly in the monolithic form^10–13^. Consequently, obtaining porosity-free TiB_2_ compounds requires high-temperature sintering procedures, which generally result in microcracks and undesirable grain growth. Furthermore, the presence of TiO_2_ and B_2_O_3_ layers on the surface of TiB_2_ is unfavorable for the consolidation of the contemplated ceramic^14–17^. To circumvent these limitations, several practical strategies have been introduced in the literature^18^. For example, replacing the conventional powder metallurgy process with advanced techniques such as pulsed electric-current sintering, also known as spark plasma sintering (SPS), could be a plausible solution for manufacturing desirable ceramics^19–22^. The SPS technique has the advantage of external pressure compared to the sintering method, which noticeably decreases both the required dwelling time and sintering temperature^23–26^. Accordingly, the ceramics produced via the former method offer a finer microstructure, lower residual porosity, and superior mechanical features compared to their counterparts sintered using conventional methods^27–29^. In addition, introducing booster second phases comprising SiC, TiC, and B_4_C*,* as reinforcement or sintering aids has been reported to be beneficial for the sinterability and quality enhancement of TiB_2_-based composites^30–33^. The incorporation of a suitable second phase may result in the in-situ formation of nanosized phases, which significantly affects the final mechanical properties, especially the fracture toughness and strength^34–37^.

Among the examined ingredients used as additives for the preparation of TiB_2_-based ceramics, SiC has consistently shown reliable improvement owing to its ability to supplement high-temperature oxidation resistance, grain refining, fracture toughness, and flexural strength^38^. Yan et al.^39^ prepared TiB_2_–SiC composites comprising varying amounts of SiC of different morphologies (whiskers and particles). The composites were produced via hot-press sintering at 25 MPa and 1950 °C for 120 min. According to the acquired results, the composite having a higher SiC content presented higher values of relative densities compared to the other composites. However, considering the composites introduced by SiC whiskers, all the ceramics reached their expected completely dense state. Moreover, X-ray diffraction analysis (XRD) and field emission scanning electron microscopy (FESEM) results confirmed the in-situ generation of TiC and graphite during the sintering process, owing to which, chemical interactions occurred among the SiC phases and the oxide impurities existing in the system. Lin et al.^40^ studied the effect of the addition of carbon nanotube (CNTs) on the microstructure and mechanical properties of SPSed TiB_2_–SiC ceramics. No in-situ phase could be detected in the XRD patterns of the samples sintered at 1600–1800 °C, which indicates the low reactivity of the system under the applied conditions. In addition, a sintering temperature of 1750 °C and CNTs content of 15 vol% were found to be the optimum parameters for reaching the highest fracture toughness and flexural strength values of ~ 10.4 MPa.m^1/2^ and 925 MPa, respectively. In another interesting study, Vajdi et al.^41^ prepared a TiB_2_–SiC composite comprising 2 wt% graphene nanoplatelets (GNPs). The sintering process was achieved at 40 MPa and 1800 °C for 7 min using the SPS technique, which resulted in a ceramic with a relative density of 96% and two in-situ ingredients of B_4_C and TiC. The in-situ compounds were mainly formed owing to the reduction of TiB_2_ surface impurities owing to the added GNPs. Fei et al.^42^ investigated the role of short carbon fiber (C_sf_) reinforcement on the densification and mechanical properties of TiB_2_/C composite. The C_sf_ additive not only enhanced the densification behavior of TiB_2_, but also increased the mechanical features and particularly the fracture toughness of the prepared composite, reaching the peak value of 3.61 MPa m^1/2^ when 4 wt% C_sf_ was incorporated into the composite. The properties enhancement was achieved due to the activation of various toughening mechanisms in the presence of C_sf_.

**Figure 1:**
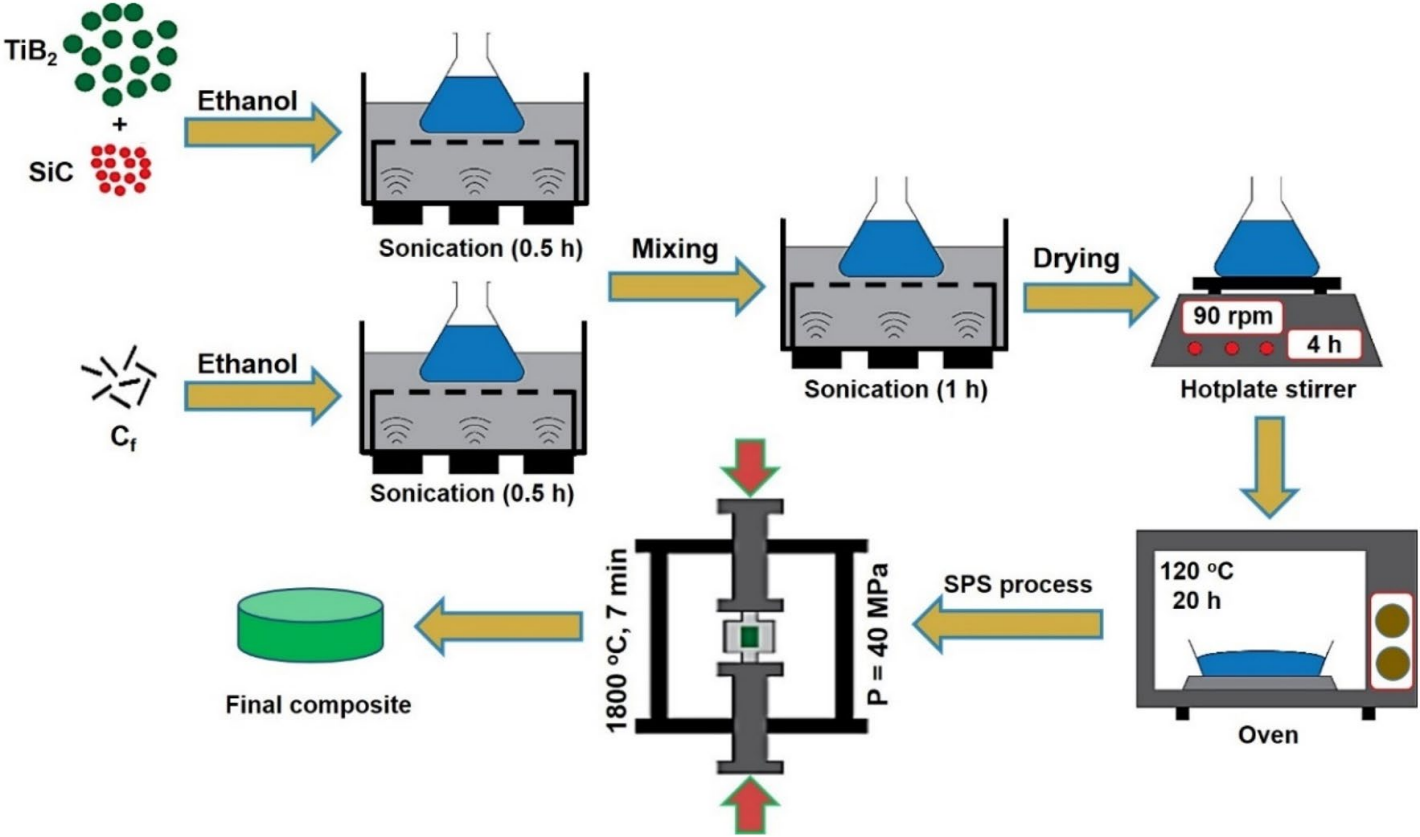
Preparation procedure of C_f_-incorporated TiB_2_–SiC composite.

This research introduces the preparation of a TiB_2_-25 vol% SiC composite containing 2 wt% carbon fibers (C_f_) utilizing the constructive role of SiC and carbon fiber additives on the sinterablity and mechanical properties of the prepared composite. The overall preparation procedure of the composite is demonstrated in Fig. 1. We have also carefully evaluated the possibility of the formation of in-situ phases, including TiC and B_4_C, using complementary characterization techniques, *e.g.*, X-ray photoelectron spectroscopy (XPS) and high-resolution transmission electron microscopy (HRTEM). In addition, the results presented in this work can be compared to those in some similar studies^5,43–47^.

## Material and methods

### Preparation method

Commercially available hexagonal raw materials of TiB_2_, SiC, and C_f_ were used in this study. Table 1 summarizes the information on these materials. To obtain the desired composites (TiB_2_–25 vol% SiC–2 wt% C_f_), 13, 3.08, and 0.33 g of the powders were carefully weighed using a digital balance. The C_f_ powder was dispersed in ethanol for 30 min in an ultrasonic bath. The same process was performed to mix the TiB_2_ and SiC. The obtained TiB_2_–25 vol% SiC slurry was ball-milled for 90 min at 120 rpm using zirconia cup/balls as milling tools. Then, the ethanol-dispersed C_f_ slurry was added to the ball-milled TiB_2_–SiC mixture and ultrasonicated for 60 min. Subsequently, the resulting mixture was heated using a hotplate stirrer for 4 h to evaporate the ethanol medium. Complete dehumidification was achieved at 120 °C for 20 h in a universal oven. The dried admixture was loaded into a graphite mold (25-mm thickness and 15-mm diameter) and sintered at 40 MPa and 1800 °C for 7 min using an SPS apparatus. After the sintering process, the specimen was gradually cooled inside the SPS instrument before being polished out from the graphite die (the final thickness of the as-sintered composite was 3.4 mm).

**Table 1:** Technical information of the as-received powders.

Starting powders	Size	Purity (%)
TiB_2_	< 8 µm	98.0
SiC particles	< 3 µm	99.0
Carbon fibers	Diameter ~ 5 μm	99.0
(C_f_)	Length ~ 2 mm	

### Characterizations

The Archimedes principle and rule of the mixture were used to determine the bulk density and theoretical density of the SPS-treated composite, respectively. A phase study was conducted using an XRD diffractometer (D8 Advance, Bruker). Micrographs were obtained using an FESEM (SUPRA 55VP, Zeiss, Germany) equipped with an energy-dispersive X-ray spectroscopy (EDS) detector. Complementary microstructural studies were performed using HRTEM (JEOL, JEM-2100F). The sample preparation for the HRTEM studies was realized using a focused ion beam (FIB, Helios G4 Thermo Fisher Scientific). Elemental distribution analysis was performed using a field-emission electron probe micro-analyzer (FEEPMA, JXA-8530F JEOL). XPS analysis was carried out using an Al Kα source (VG Scientifics, Sigma probe). For peak separation, the Fityk software (Ver. 1.3.1, Marcin Wojdyr) was used^48^. Computer programs, namely HSC (Ver. 6, Outokumpu Research Oy) and Gatan microscopy suite (Ver. 2.1114040, Gatan Inc.), were used to assess the chemical reactions and analyze the HRTEM images, respectively. To measure the mechanical characteristics of the composite, the nanoindentation method (Agilent G200, USA) was used with a Berkovich indenter comprising a three-sided pyramid tip. A load–displacement curve was plotted for each indentation (six indentations on average for each phase), and the relevant mechanical characteristics were extracted. The maximum load applied, holding time, and loading rate were 400 mN, 5 s, and 40 mN/S, respectively. The corresponding hardness value at any point was determined using the Oliver–Pharr technique (Eq. (1)).

1 $$H = \frac{{P_{\max } }}{{A_{c} }}$$

2 $$A_{c} = f(h_{c} ) = 24.56 \times h_{c}^{2} + 0.562 \times h_{c} + 0.003216$$

3 $$h_{c} = h_{t} - \frac{\varepsilon }{S} \times P_{\max }$$

In Eq. (1), H, P_max_, and A_c_ are the hardness, maximum load, and projected indentation area, respectively. Furthermore, h_c_ is the contact depth, which can be calculated using Eq. (3). The value of ɛ is 0.75 for the present indenter, h_t_ is the indenter displacement at maximal load, and S represents the stiffness. In addition, the elastic modulus can be obtained using Eqs. (4) and (5).

4 $$S = \frac{dp}{{dh}} = \delta \frac{2}{\sqrt \pi }E_{m} \sqrt {A_{c} }$$

5 $$\frac{1}{{E_{m} }} = \frac{{1 - \nu_{s}^{2} }}{{E_{S} }} + \frac{{1 - \nu_{i}^{2} }}{{E_{i} }}$$

where ʋ_i_ and ʋ_s_ are the Poisson’s coefficient of the indenter and sample, respectively. E_m_, E_i_, and E_s_, respectively, represent the modified elastic modulus and the elastic modulus of the indenter and specimen. Finally, δ is a constant (1.034) that depends on the indenter geometry.

## Results and discussion

FESEM micrographs of the TiB_2_ and SiC precursors and their corresponding XRD patterns can be found in our previously published study^30^. Their morphologies show that both powders comprise irregular particles with sharp edges. Moreover, they are non-uniform and have a wide range of particle sizes. In the XRD patterns, only peaks associated with hexagonal TiB_2_ and SiC can be observed, indicating the low content of possible impurities in the precursors. These results are in agreement with the information provided by the supplier, as summarized in Table 1. Although no oxide impurities were detected in the precursors, it is well known that the surfaces of TiB_2_ and SiC particles are naturally covered in some impurities. TiO_2_ and B_2_O_3_ are considered the main species available on the TiB_2_ powders^49^. However, in the case of SiC, the main surface impurity is SiO_2_. Among the aforementioned oxides, B_2_O_3_ plays a governing role in the densification behavior of TiB_2_ ceramics. B_2_O_3_ is an amorphous phase that melts at ~ 510 °C and evaporates at ~ 1860 °C. Considering that the SPS process is performed under vacuum conditions, the evaporation of this oxide occurs at approximately 1350 °C^50,51^. Indeed, while all the B_2_O_3_ cannot be volatilized at this temperature, a small portion may remain in the system. Based on the experimental results, a thin layer of B_2_O_3_ could tolerate high temperatures up to 1950 °C^51^. However, the local temperature may dramatically increase on the surface of the particles because of the sparking phenomenon^52^. As a result, it is highly possible that the evaporation and condensation of B_2_O_3_ occurred immediately after the initiation of the SPS process. Thus, both liquid and gaseous forms of B_2_O_3_ were available throughout the sintering process. Furthermore, B_2_O_3_ can generate low-melting-point eutectic phases with both TiO_2_ and SiO_2_, resulting in the formation of more liquid phases in the system^53–55^. Owing to the presence of the carbonaceous phase, i.e., C_f_, this molten phase can be reduced to non-oxide ingredients, as specified below.

After sintering, the relative density of the ceramic was calculated as described in the experimental section. According to the estimation, incorporating 2 wt% C_f_ into TiB_2_–25 vol% SiC resulted in a composite with a relative density of 98.5%. On comparing this value with that of the C_f_-free sample sintered under similar conditions^45^, it can be inferred that the introduction of C_f_ could play an important role as a sintering aid for TiB_2_–SiC preparation. Torizuka et al.^56^ reported a relative density of 99% for TiB_2_–2.5 wt% SiC SPSed at 1600 °C. Furthermore, Vajdi et al.^41^ added 2 wt% graphene nanoplatelets to TiB_2_–25 vol% SiC, realizing a relative density of 96% using the SPS technique at 1800 °C. To understand how the addition of 2 wt% C_f_ could enhance the consolidation behavior, the TiB_2_–SiC system should be carefully studied, particularly in terms of densification mechanisms and reactivity.

**Figure 2:**
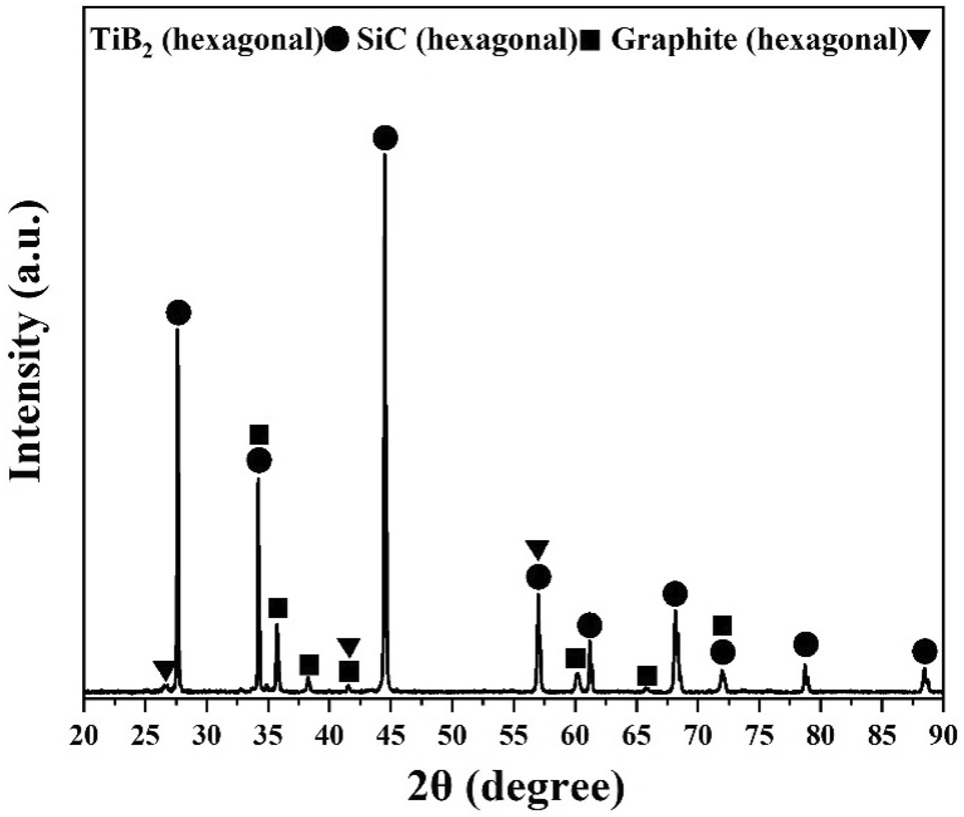
XRD pattern of C_f_-incorporated TiB_2_–SiC composite.

To clarify what happens during the sintering process, the SPSed composite was initially studied using an XRD analysis. The acquired XRD pattern (Fig. 2) was subsequently assessed with respect to the most probable crystalline phases, which resulted in the identification of the following compounds: TiB_2_ (crystalline hexagonal structure, reference code 01–075–0967, space group P6/mmm), SiC (hexagonal crystalline structure, reference code 01–073–1663, space group P63mc), and graphite-2H (hexagonal crystalline structure, reference code 00–041–1487, space group P63/mmc). The fact that no new phase could be identified in this composite implies the non-reactivity of the system. However, the possibility of the progress of some minor reactions still exists, especially with the participation of oxide impurities. It is worth mentioning that a eutectic phase can be formed in the TiB_2_–SiC system with 52 wt% SiC at 2190 °C. However, no liquid phase was expected from this source under the current sintering conditions^50^. In contrast, both TiO_2_–B_2_O_3_ and SiO_2_–B_2_O_3_ can form low-melting eutectic phases, which facilitate mass transfer during the sintering process. The most likely chemical interactions among the existing ingredients in the TiB_2_–SiC–C_f_ system are presented in Eqs. (6)–(8). Based on Eqs. (6) and (7), the TiO_2_ and B_2_O_3_ compounds can be reduced by C_f_ and SiC, which results in the in-situ formation of TiB_2_ and some gaseous phases^57^. These reactions were also assessed using the HSC chemical package in their feasibility studies under applied SPS conditions. As a result, ΔG° at 1800 °C was calculated to be − 331 and − 278 kJ for these equations, respectively, thus confirming their favorability at the ultimate sintering temperature under standard conditions. Although both C_f_ and SiC can eliminate the oxide phases, C_f_ is a stronger reductant than SiC. The C_f_ addition can also act as a strong reducing agent for SiO_2_ impurities. As shown in Eq. (8), SiO_2_ is reduced by C_f_, forming in-situ SiC and a gaseous phase. Moreover, this reaction was studied using the HSC program and confirmed as a possible scenario in the sintering system (ΔG° at 1800 °C ~–90 kJ). The formation of such in-situ phases is highly important for ceramic-based composites. This phenomenon promotes solid diffusion and forms fresh fine particles with high activity, resulting in high relative densities and a strong binding of the matrix^58^.

6 $$TiO_{2} + B_{2} O_{3} + \, 5C_{f} \to \, TiB_{2} + \, 5CO_{(g)}$$

7 $$2TiO_{2} + 2B_{2} O_{3} + 5SiC \to 2TiB_{2} + 5CO_{(g)} + 5SiO_{(g)}$$

8 $$SiO_{2} + \, 3C_{f} \to \, SiC \, + \, 2CO_{(g)}$$

**Figure 3:**
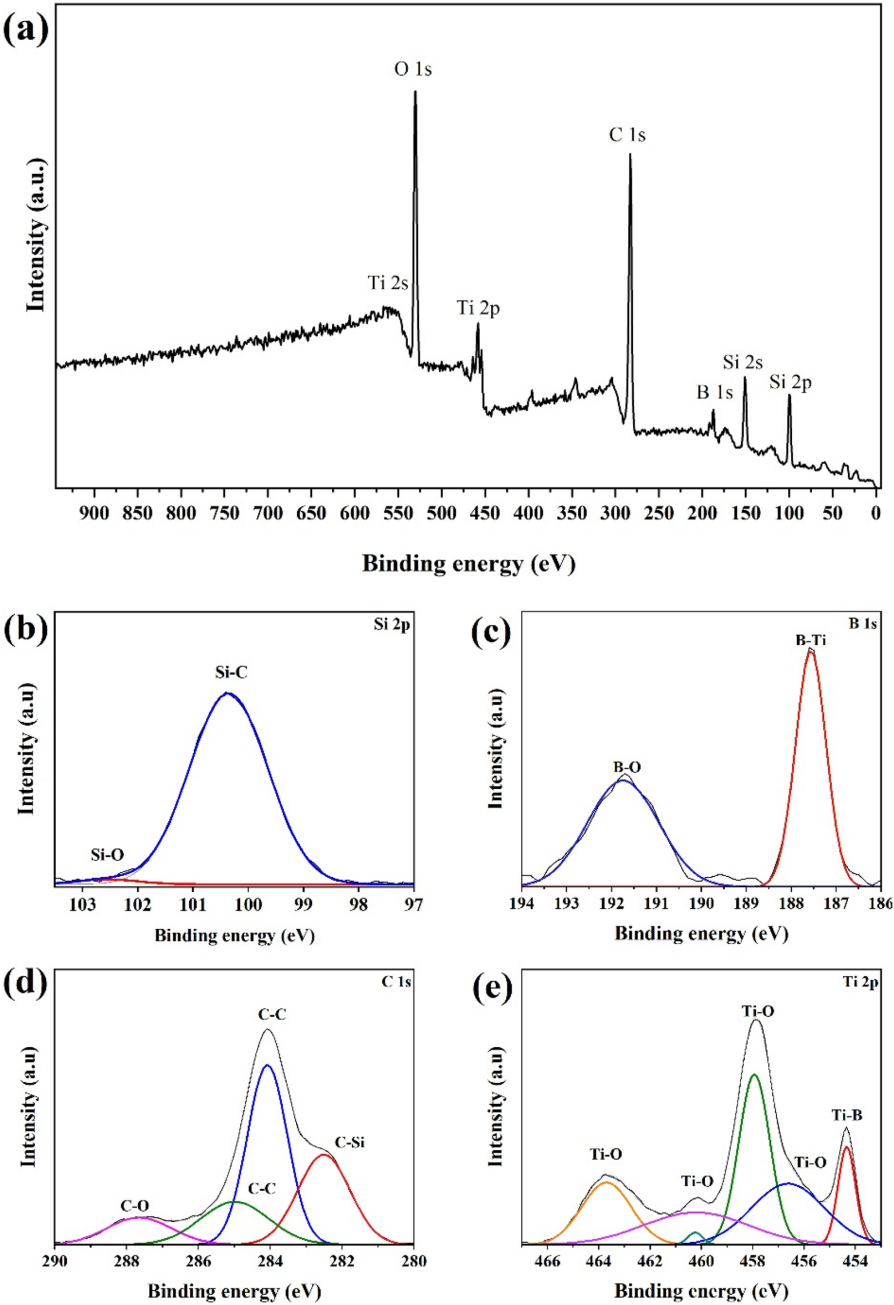
XPS analysis of C_f_-incorporated TiB_2_–SiC composite (**a**) survey, (**b**) Si 2p, (**c**) B 1s, (**d**) C 1s, and (**e**) Ti 2p.

Although no new phase could be observed in the XRD pattern, the in-situ generation of TiC and B_4_C has been reported in similar studies^41,58^. Equations (9)–(10) present plausible reactions in which such phases can be generated^45,58^. A negative ΔG° at 1800 °C indicates the feasibility of both reactions under the present sintering conditions. Accordingly, the probable presence of these ingredients in the final microstructure should be further studied. Therefore, the SPSed sample was investigated using XPS, and the results are presented in Fig. 3. Interestingly, no peaks related to the Ti–C and B–C bonding energies could be identified in the relevant spectra. As a result, it can be concluded that the formation of TiC and B_4_C compounds was hindered by other favorable reactions; these in-situ phases were consumed after being generated. Wu et al.^58^ suggested Eq. (11) as a representation of this process, wherein TiC and B_4_C react together to realize the in-situ formation of TiB_2_ and graphite.

9 $$2TiO_{2} + 3SiC \to 2TiC + 3SiO_{(g)} + CO_{(g)}$$

10 $$2B_{2} O_{3} + 7C_{f} \to B_{4} C + 6CO_{(g)}$$

11 $$2TiC + B_{4} C \to 2TiB_{2} + 3C$$

**Figure 4:**
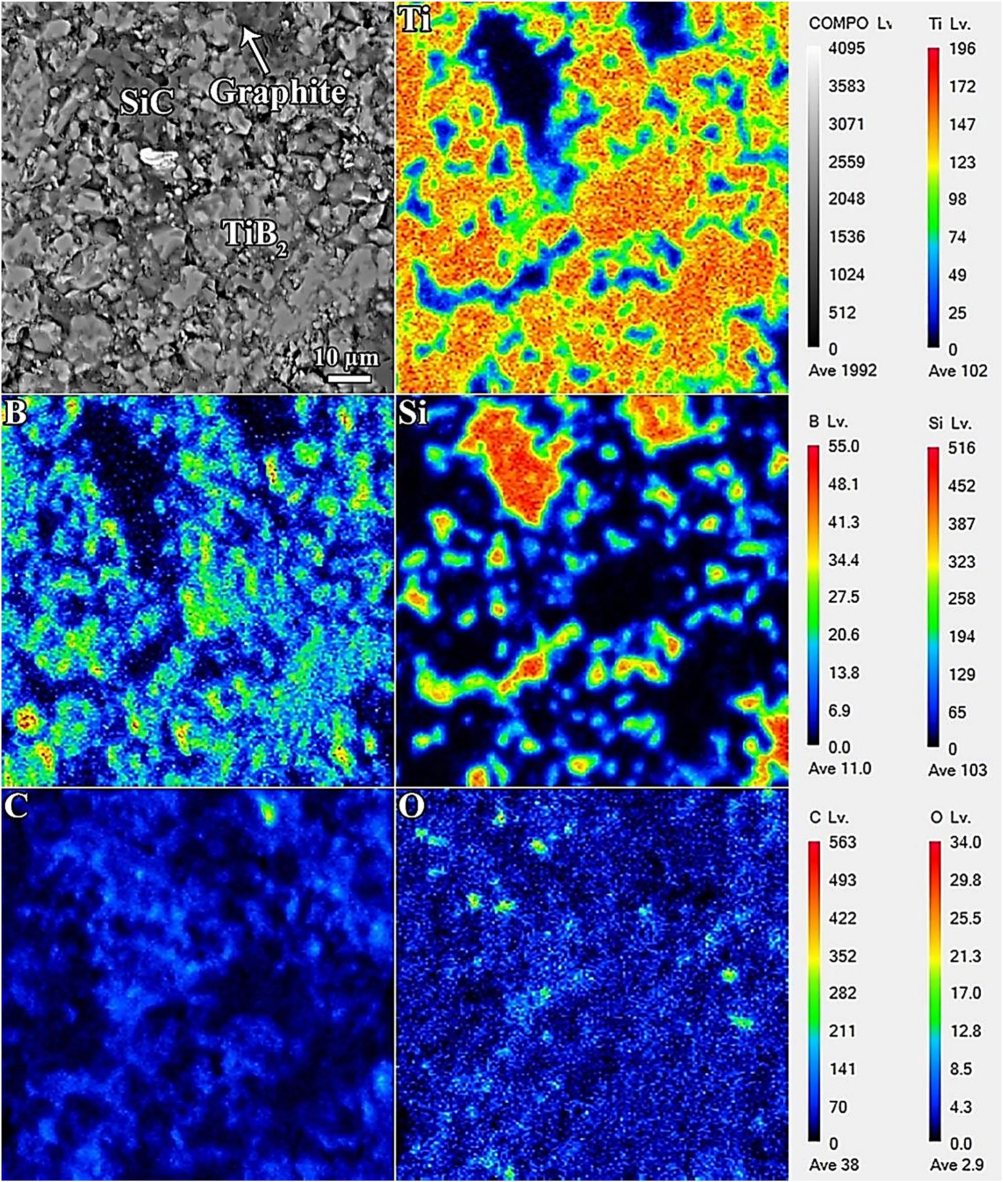
FESEM image of the fracture surface of C_f_-incorporated TiB_2_–SiC composite, and the relevant FEEPMA elemental mapping of Ti, B, Si, C, and O elements.

**Figure 5:**
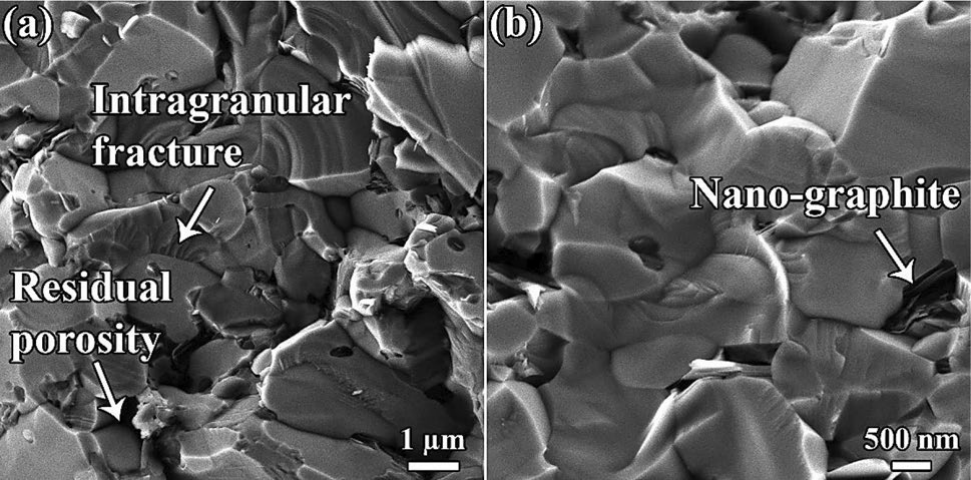
(**a** and **b**) FESEM images of C_f_-incorporated TiB_2_–SiC composite in different magnifications.

**Figure 6:**
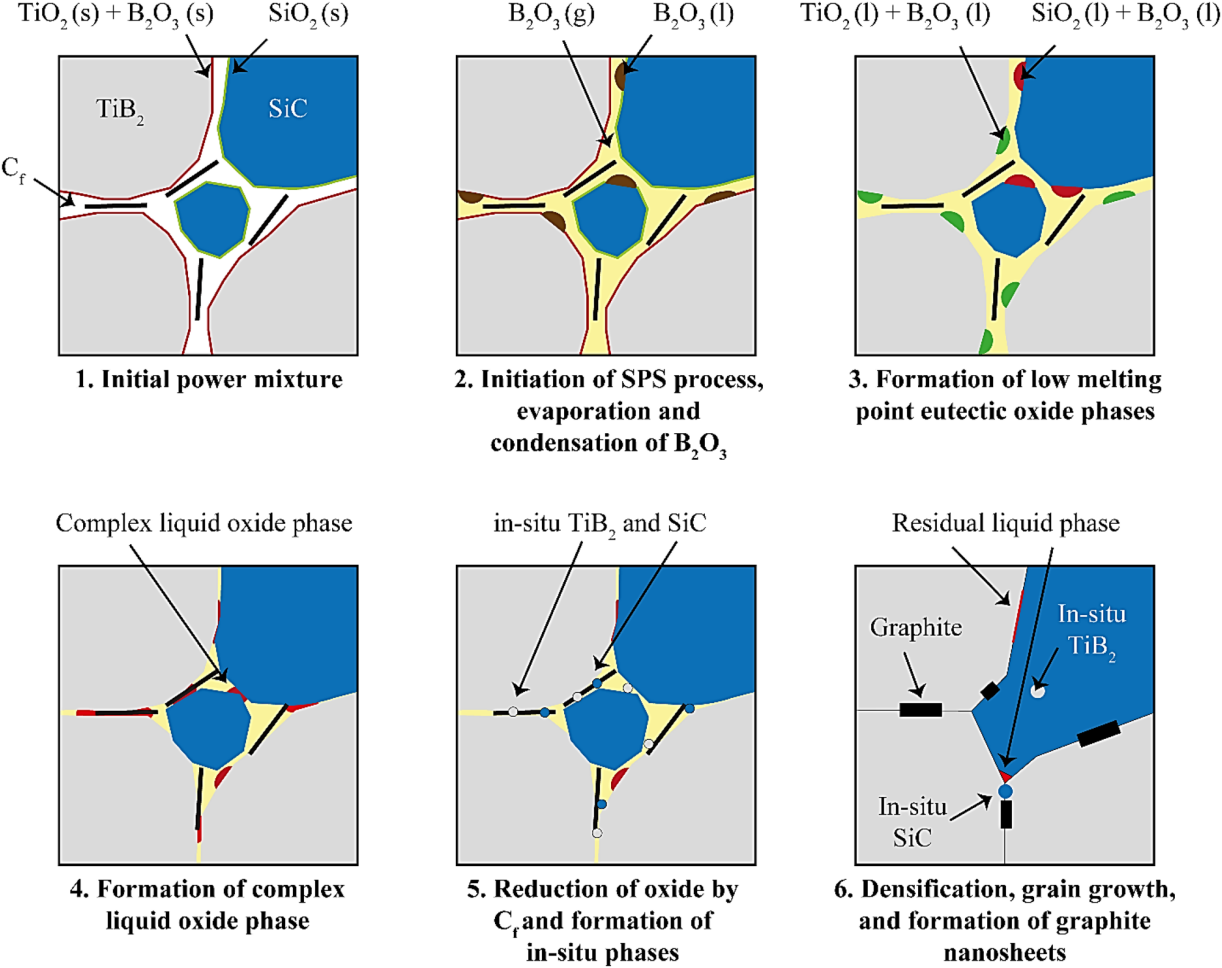
Schematic of steps for the sintering of C_f_-incorporated TiB_2_–SiC composite, which results in the final microstructure.

The microstructures of the samples were examined for both the fracture and polished surfaces. Figure 4 presents the backscattered FESEM image of the polished surface of the TiB_2_–SiC composite comprising added C_f_ and the corresponding FEEPMA map images. The uniform distribution of both the SiC reinforcement and carbonaceous phase in the TiB_2_ matrix is apparent. Moreover, according to the fractographs presented in Fig. 5, it can be observed that the final microstructure contains a low content of residual porosity. Thus, the densification progressed during the SPS process, which agrees with the value of relative density reported earlier. As previously discussed, the role of liquid-phase sintering is limited in this sintering system because of the low oxide impurity content. However, removing them by the advancement of some chemical interactions can significantly improve the sintering behavior of TiB_2_. Furthermore, it should be taken into consideration that the vaporization and condensation of B_2_O_3_ play a noticeable role in controlling the grain growth^59^. The residual liquid phase in the final composite fills some of the remaining pores because of the capillary force, thereby improving the relative density of the obtained composite^51^. The phenomena associated with oxide impurities and liquid-phase sintering are schematically illustrated in Fig. 6. Zhang et al.^60^ divided the SPS process of TiB_2_-based materials into three steps: (I) particle activation, (II) particle contact and connection, and (III) fast consolidation. Owing to the low self-diffusion coefficient of TiB_2_, no apparent changes were observed in the particles at low temperatures. Nevertheless, on reaching the sintering temperature of 1400 °C, neck formation occurs owing to the interruption of the surface oxide. Thus, the majority of TiB_2_ particles were activated in this phase. When the sintering temperature increased, densification accelerated owing to the cumulative effects of vaporization and condensation. The sintering temperature (1800 °C) is an important point at which rapid densification occurs. In comparison, TiB_2_ can reach a relative density of ~ 98% at 1800 °C, whereas this value is ~ 78% at a sintering temperature of 1500 °C.

Considering the morphology of the carbonaceous phase in the final microstructure (Fig. 5), C_f_ could not retain its initial morphology during the sintering process. In the case of C_f_, some graphite nanosheets nucleated and grew in the microstructure, particularly at the grain boundaries and triple junctions. In terms of the fracture mode, both intergranular and intragranular fracture types can be observed in the fractographs (Fig. 5). When a crack propagates through grain boundaries instead of grain domains, it deteriorates the fracture toughness because of the high energy consumption^58^. The difference between the thermal expansion coefficients of TiB_2_ (8.1 × 10^−6^ K^-1^) and SiC (4.7 × 10^−6^ K^-1^) is mainly responsible for this type of fracture^51^. However, when a crack reaches a large TiB_2_ particle, there is a strong possibility of it passing through, thus forming a smooth fracture surface. Furthermore, it can be observed in some areas that the fracture surface has a step-shaped morphology. According to the fracture mechanics of ceramic materials, the force that drives the spread of cracks diminishes if the fracture consumes the energy of the crack stress field, thus resulting in an improved toughness^51^.

**Figure 7:**
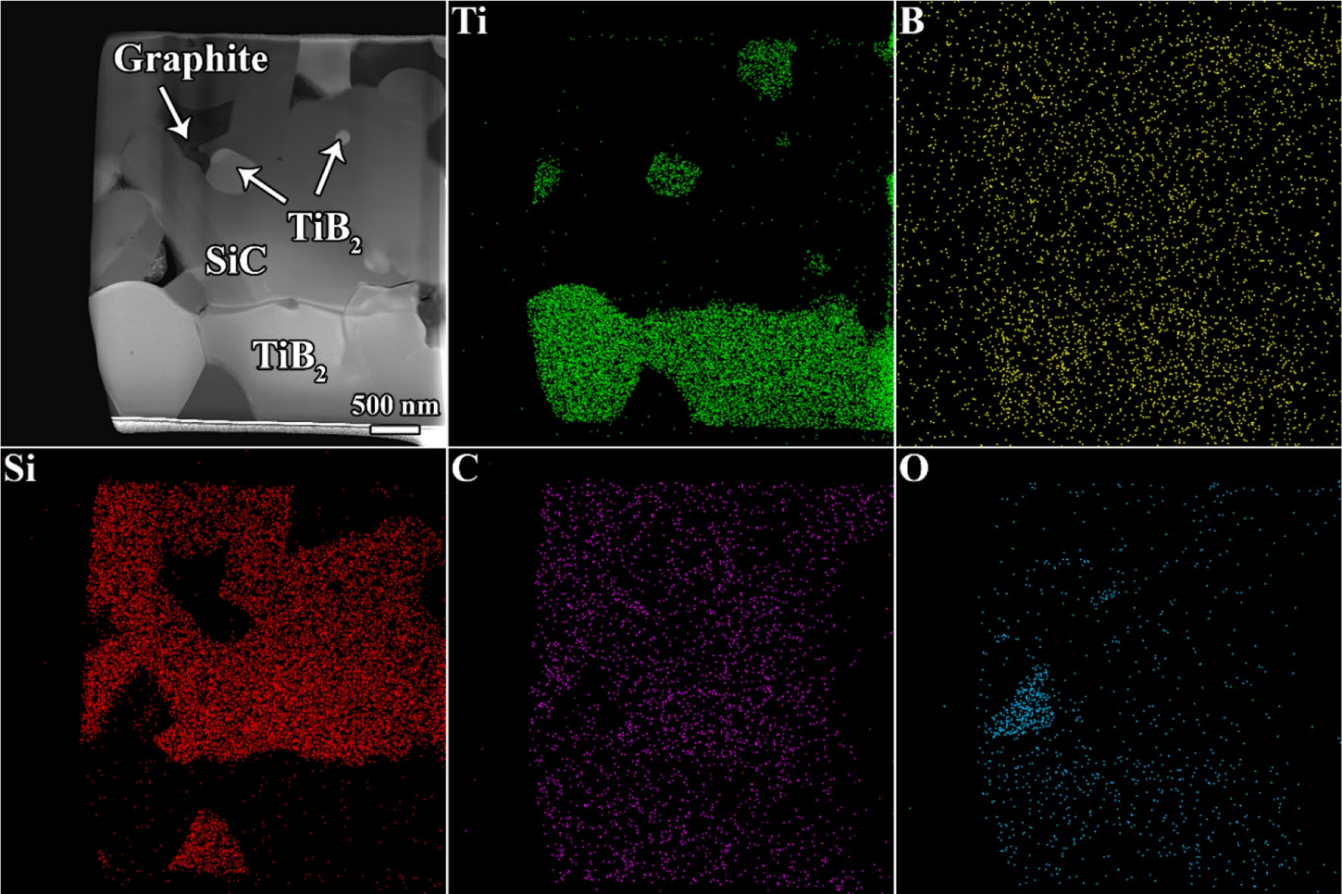
STEM image of the FIB specimen of the C_f_-incorporated TiB_2_–SiC composite, along with the relevant EDS maps of Ti, B, Si, C, and O elements.

Figure 7 presents a scanning transmission electron microscope (STEM) micrograph of the prepared FIB sample of the TiB_2_–SiC composite comprising C_f_ along with the corresponding EDS map images. It can be inferred that the bright-color, grey-color, and dark-color phases are associated with the TiB_2_, SiC, and graphite compounds, respectively. Except for the oxygen-rich phase shown in the STEM image, no other ingredient can be observed in the EDS mapping. This observation is also in agreement with the previous XRD, XPS, and FEEPMA results. In the following, the FIB sample is discussed in greater detail, particularly with respect to the interfacial areas, using the TEM and HRTEM techniques.

**Figure 8:**
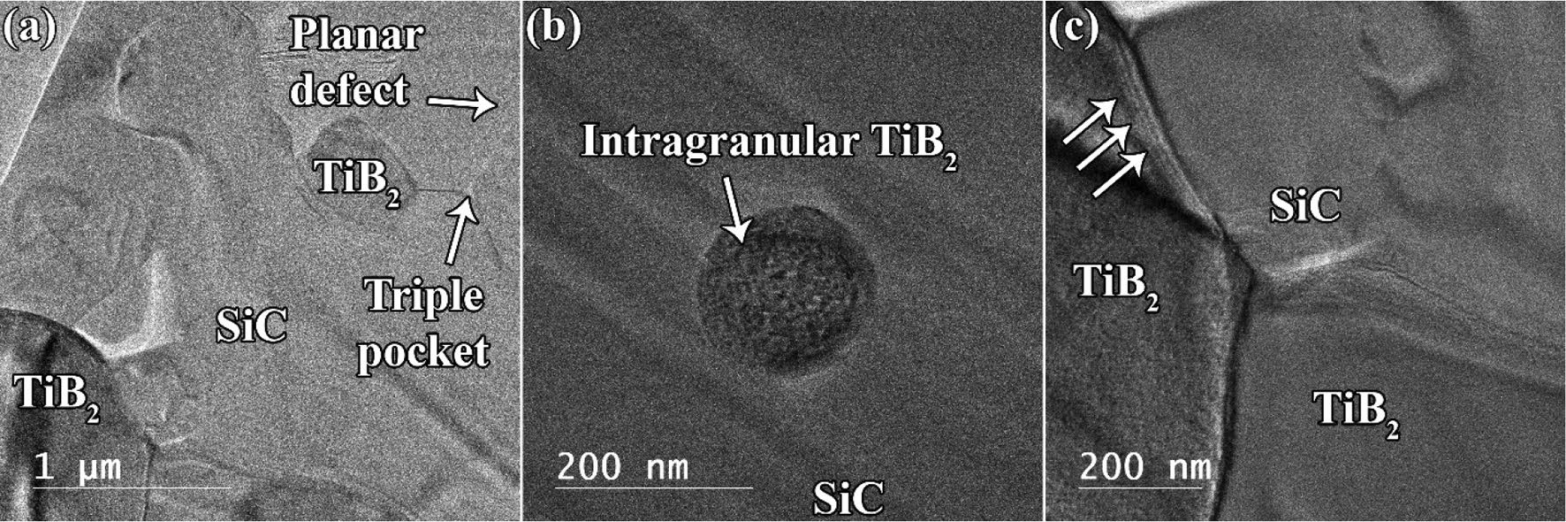
TEM images of the C_f_-incorporated TiB_2_–SiC composite showing (**a**) TiB_2_ and SiC phases, (**b**) an intergranular TiB_2_ particle, and (**c**) the interface of TiB_2_/SiC.

Figure 8a demonstrates that the densification progressed appropriately, and some porosity-free interfaces were formed at both the SiC/TiB_2_ and SiC/SiC interfaces. Moreover, a triple pocket can be observed among the three SiC particles, which may be filled with a residual liquid phase. Monteverde et al.^61^ introduced the convention phenomenon as the driving force for drawing the liquid phase out of the grain boundaries and driving it to triple pockets and the residual porosity. This happens owing to the formation of a gradient in the surface tension of various molten phase zones of different compositions. As shown in Fig. 8a, a planar defect can be observed in the SiC phase, which is possibly generated owing to a mismatch between the coefficients of thermal expansion of this phase and the TiB_2_ matrix. The availability of an intragranular TiB_2_ phase in SiC (Fig. 8b) verifies the in-situ formation of ultrafine TiB_2_ and SiC grain coarsening. TiB_2_ particles were formed in-situ as a result of TiO_2_ reduction; however, they were entrapped between two adjacent SiC particles, which coalesced and formed a larger grain. This phenomenon is graphically illustrated in Fig. 6. Considering the TiB_2_/SiC interface (Fig. 8c), the boundary line is smooth on the SiC side, while some non-uniformity can be observed on the TiB_2_ side (indicated by three white arrows). Such a boundary was formed because of the vacancy concentration. Vacancies are typically formed by atomic diffusion. However, in the SPSed specimens, empty atomic positions can be formed because of the evaporation of the surface atoms between two adjacent particles in the initial step of the SPS during plasma formation at high temperatures^62^.

**Figure 9:**
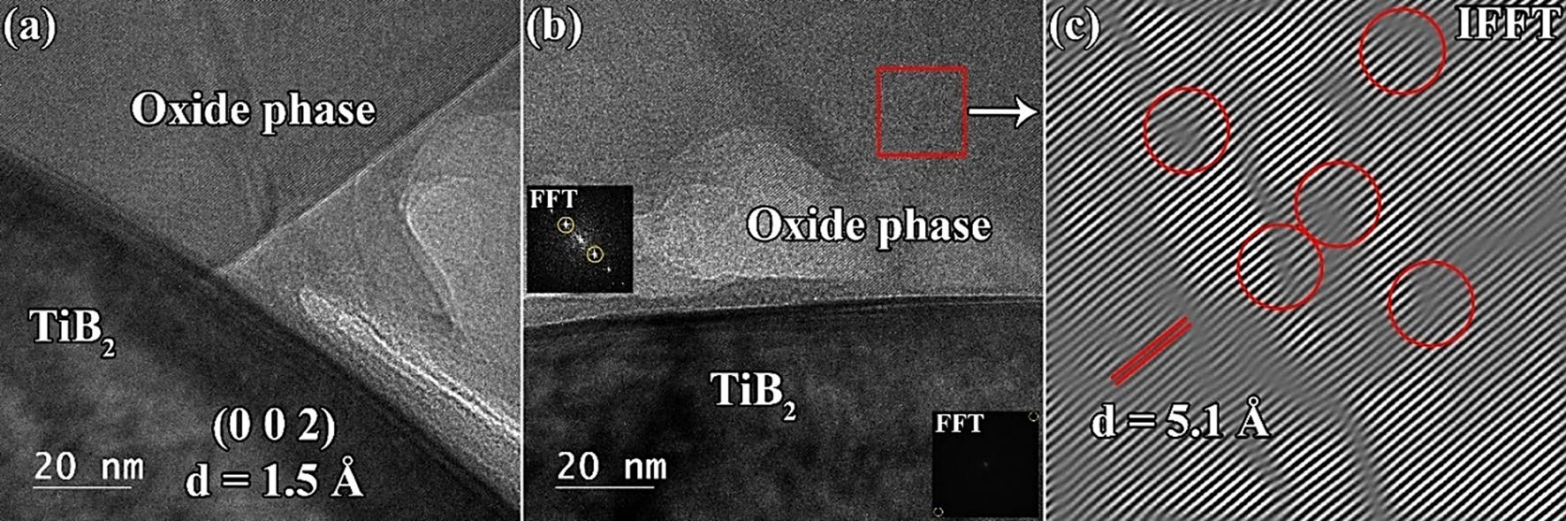
(**a** and **b**) HRTEM images of C_f_-incorporated TiB_2_–SiC composite showing a TiB_2_/residual oxide interface, and (**c**) the corresponding IFFT analysis.

The interfacial areas of TiB_2_ and oxygen-rich compounds are presented in Fig. 9. The TiB_2_ phase represents the (0 0 2) crystalline plane with a d-spacing of ~ 1.5 Å. The boundary non-uniformity line on the TiB_2_ side can be observed in the HRTEM image, which implies atomic diffusion. In contrast, the interfacial area in the oxide phase appears to comprise a crystalline structure. The pertaining fast Fourier transform (FFT) and inverse FFT (IFFT) from this oxide phase (red square) are presented in Fig. 9b, c. In the IFFT image in Fig. 9c, several dislocations and disordered atomic planes can be observed. Apart from the crystalline defects, the d-spacing of this phase was calculated to be ~ 5.1 Å, which does not match any of the available components in the system. This phase is perhaps associated with ZrO_2_ originated during the ball milling process of the precursors.

**Figure 10:**
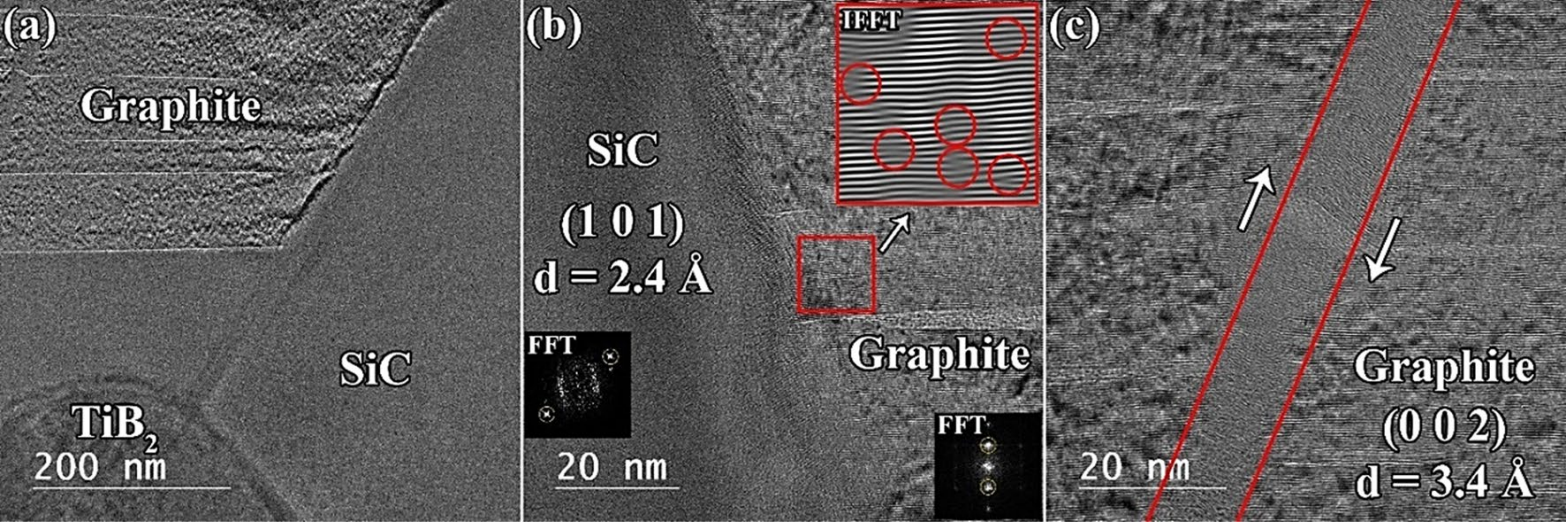
(**a**) TEM image of C_f_-incorporated TiB_2_–SiC composite showing TiB_2_, SiC, and graphite compounds. (**b**) HRTEM of the SiC/graphite interface. (c) HRTEM of the deformed graphite nanosheets.

The morphology of the graphite in the SPSed sample is presented in Fig. 10. As noted previously, C_f_ could not maintain its initial morphology and was converted into graphite nanosheets. Figure 10b shows the interface between the (1 0 1) plane of SiC (d-spacing of 2.4 Å) and the (0 0 2) plane of graphite (d-spacing of 3.4 Å). The absence of an apparent boundary between these two phases can be attributed to the role of carbon in reducing SiO_2_ and the formation of ultra-fine SiC particles. When this effect is combined with liquid-phase sintering, it can form very strong interfaces. Graphite nanosheets were also studied using FFT and IFFT techniques (Fig. 10b), which revealed the presence of dislocations, atomic plane distortions, and atomic plane disorders in their crystalline structures. Moreover, the graphite phase experienced a shear strain, which deformed it, as shown in the HRTEM image (Fig. 10c). Similar to the previous cases, it appears that a mismatch between the coefficients of thermal expansion of graphite and other ingredients (SiC in this case) is responsible for such deformation during the cooling stage.

The hardness values of TiB_2_ and SiC and their interfacial areas were measured via nanoindentation tests. The results are presented in Fig. S1 and Table 2. Some FESEM images of indentation areas are shown in Fig. S2. It should be mentioned that the numbers presented in Table 2 are the mean values of six indentation tests. All the load–displacement curves illustrated in Fig. S1 fall under three zones: “ascending”, “linear”, and “descending”. The calculation of the hardness using the Oliver–Farr method results in data propagation owing to the surface roughness^50,51^. When the routine technique is used to measure the penetration depth, the hardness changes significantly in areas near the surface. For instance, the slippage of the indenter’s tip may occur at protrusions at low loads. As a result, the obtained value for the indentation depth and consequently the projected area would be greater than those on flat surfaces or surfaces comprising depressions, which results in lower hardness values. In summary, even slight wrinkles may result in different hardness values related to a single phase. As presented in Table 2, the sequence of the phases in terms of elastic modulus, hardness, and stiffness values is as follows; hardness: TiB_2_ > SiC > TiB_2_/SiC interface; elastic modulus: TiB_2_ > SiC > TiB_2_/SiC interface; and stiffness: TiB_2_ > SiC > TiB_2_/SiC interface.

**Table 2:** Stiffness, elastic modulus, and hardness of different phases in the C_f_-incorporated TiB_2_–SiC composite.

Phases	Elastic modulus (GPa)	Hardness (GPa)	Stiffness (N/m)
TiB_2_	456 ± 23	35 ± 4	1.49 ± 0.25
SiC	421 ± 27	27 ± 6	1.24 ± 0.12
TiB_2_–SiC interface	341 ± 16	23 ± 5	1.21 ± 0.20

As an intrinsic characteristic of a substance, its elastic modulus depends on its crystalline structure and atomic bonding forces. For a multi-phase composite, the overall elastic modulus can be appraised using the elastic modulus and volume fraction of each phase. The stiffness values follow a similar trend to those of the elastic modulus. The lowest mechanical values obtained for the TiB_2_/SiC interfacial areas can be attributed to the nature of such interfaces.

The plastic and elastic energies stored on the surface of the phases after the application of the indentation can be determined using the load–displacement curve. The total mechanical work (U_t_) is equal to the area under the loading curve, whereas the elastic energy (U_e_) can be measured by calculating the area under the unloading curve. With both these values, the plastic energy (U_p_) can be obtained from Eq. (12)^63,64^.

12 $$U_{t} = U_{e} + U_{p}$$

For each phase, the plasticity index (U_p_/U_t_) and elastic recovery (U_e_/U_t_), which are the two main factors controlling the mechanical behavior of a material, were calculated, as presented in Table 3 and Fig. S3. The plasticity index represents the intrinsic behavior of a compound under plastic deformation, whereas the elastic recovery indicates the resistance of a phase against impact loading^65^. As shown in Fig. S3, the most significant elastic recovery was obtained for the TiB_2_/SiC interface, whereas the highest plasticity index was obtained for the SiC reinforcement.

**Table 3:** Calculated total energy, plastic energy, elastic energy, plasticity index, and elastic recovery of different phases in C_f_-incorporated TiB_2_–SiC composite.

Phase	Total energy U_t_ (mN nm)	Plastic energy U_p_ (mN nm)	Elastic energy Ue (mN nm)	Plasticity index U_p_/U_t_	Elastic recovery U_e_/U_t_
TiB_2_	131,725	62,416	69,309	0.47	0.53
SiC	138,422	74,773	63,649	0.54	0.46
TiB_2_–SiC interface	163,235	74,322	88,913	0.45	0.55

## Conclusions

The microstructure, densification behavior, and mechanical characteristics of the C_f_-incorporated TiB_2_–SiC composite were investigated in this study. The ceramic composite was sintered at 40 MPa and 1800 °C for 7 min. It attained a relative density of 98.5%. The added carbonaceous phase could participate in the removal of surface oxides, thus transforming them into ultra-fine TiB_2_ and SiC particles. Furthermore, no C_f_ residue was found in the SPSed sample, as it transformed into graphite nanosheets. According to the micrographs, the prepared composite was fractured in a mixed mode, i.e., both intra- and intergranular. Moreover, owing to the role of the liquid-phase sintering and oxide removal, majority of the interfaces were found to be free of undesirable phases and residual porosity. Finally, the TiB_2_, SiC, and TiB_2_/SiC interfaces were studied using the nanoindentation technique to obtain the following sequence in terms of the calculated hardness, elastic modulus, and stiffness values: TiB_2_ > SiC > TiB_2_/SiC interface.

## Supplementary Information

Supplementary Information.

## Data Availability

All data generated or analyzed during this study are included in this published article.
