# Supplementary material for: Nanostructure and nanoindentation study of pulse electric-current sintered TiB2–SiC–Cf composite
Source: Sci Rep. 2023 Jan 7;13:379. doi: 10.1038/s41598-022-27186-8 (PMC9825507; doi:10.1038/s41598-022-27186-8)
Supplement: Supplementary file 1 — Supplementary Information. [file 41598_2022_27186_MOESM1_ESM.docx]

**Nanostructure and nanoindentation study of pulse electric-current sintered TiB_2_-SiC-C_f_ composite**

Mohammadreza Shokouhimehr ^1^, Seyed Ali Delbari ^2^, Abbas Sabahi Namini ^2^*, Ehsan Taghizadeh ^3^, Sunghoon Jung ^4^, Jin Hyuk Cho ^5^, Quyet Van Le ^5^, Joo Hwan Cha ^6^, Soo Young Kim ^5^*, Ho Won Jang ^1^*

^1^ Department of Materials Science and Engineering, Research Institute of Advanced Materials, Seoul National University, Seoul, 08826, Republic of Korea.

^2^ Department of Engineering Sciences, Faculty of Advanced Technologies, University of Mohaghegh Ardabili, Ardabil, Iran.

^3^ Department of Mechanical and Aerospace Engineering, University of California, Los Angeles, 420 Westwood Plaza, Los Angeles, California 90095, USA.

^4^ Advanced Nano Surface Department, Surface Technology Division, Korea Institute of Materials Science, Changwon 51508, Korea.

^5^ Department of Materials Science and Engineering, Institute of Green Manufacturing Technology, Korea University, 145, Anam-ro Seongbuk-gu, Seoul 02841, Republic of Korea.

^6^ Innovative Enterprise Cooperation Center, Korea Institute of Science & Technology, Hwarangro 14-gil, Seongbuk-gu, Seoul, Republic of Korea.

*** Corresponding authors**

A. Sabahi Namini ([sabahi@uma.ac.ir](mailto:sabahi@uma.ac.ir)), S. Y. Kim ([sooyoungkim@korea.ac.kr](mailto:sooyoungkim@korea.ac.kr)), H. W. Jang (hwjang@snu.ac.kr)


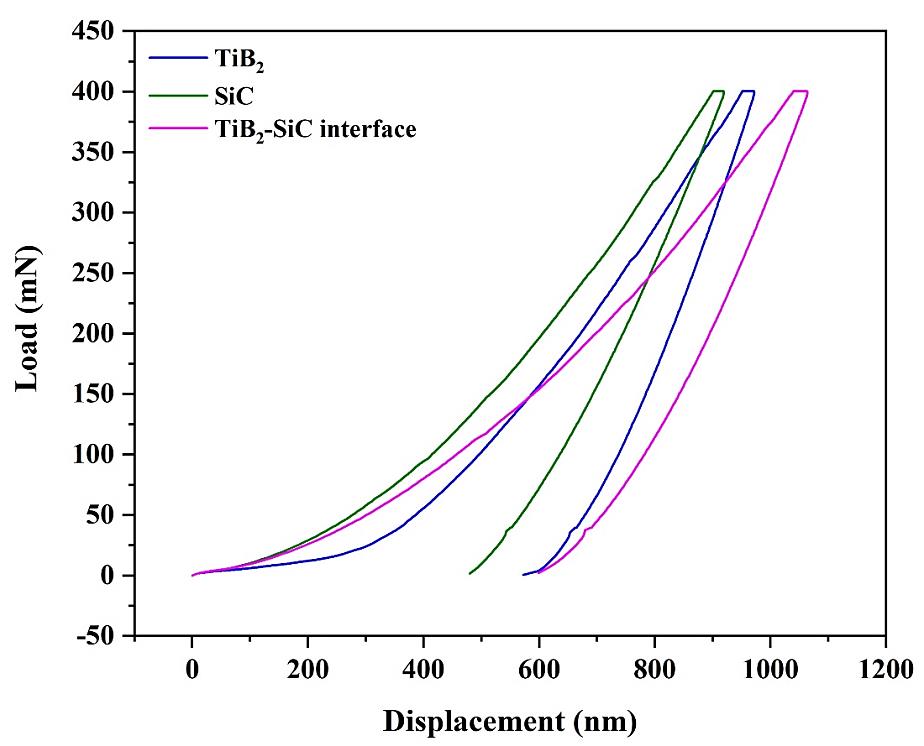


**Fig. S1.** Load–displacement curves for different phases of the C_f_-incorporated TiB_2_–SiC composite.


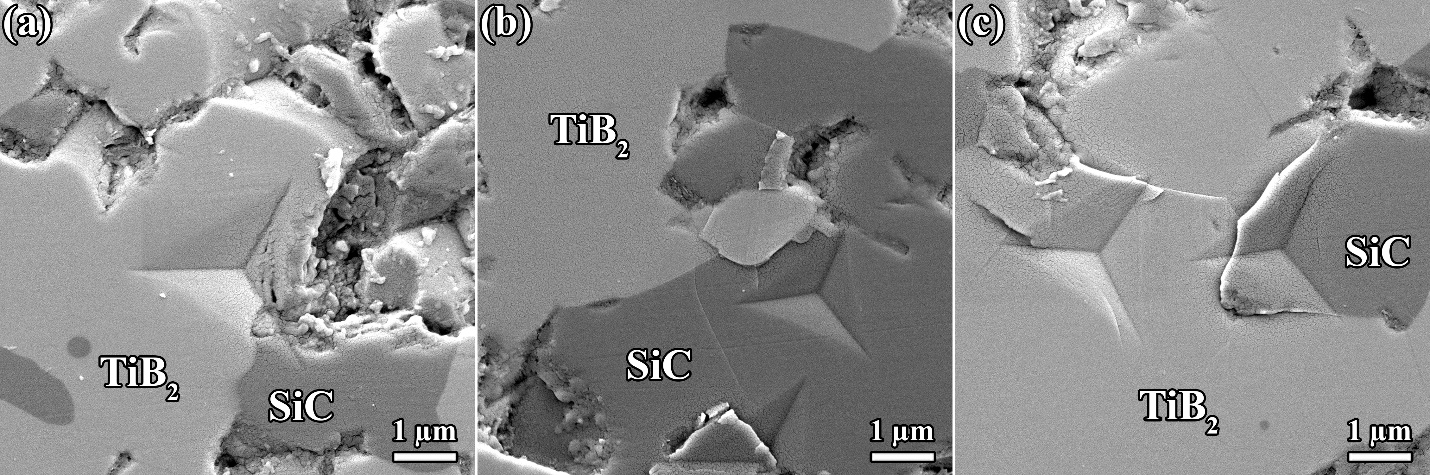


**Fig. S2.** FESEM images of load–displacement curves for different phases of the C_f_-incorporated TiB_2_–SiC composite.


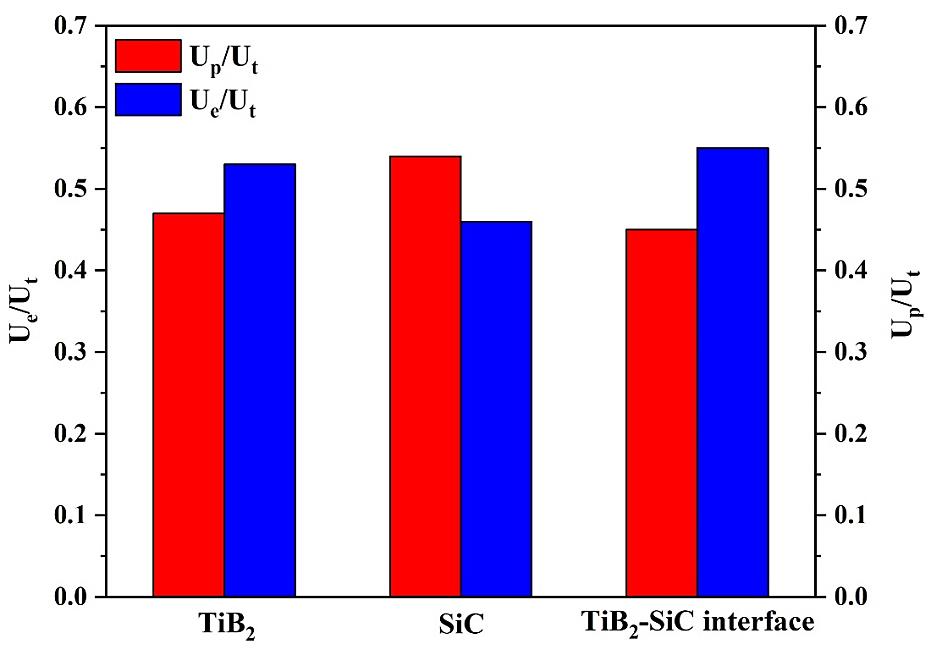


**Fig. S3.** Plasticity index and elastic recovery of different phases in C_f_-incorporated TiB_2_–SiC composite.
